# Edridge Green Lecture 2022—demystifying clinical trials and regulatory approvals in drug development

**DOI:** 10.1038/s41433-024-03520-4

**Published:** 2024-12-05

**Authors:** Victor Chong

**Affiliations:** 1 UCL Institute of Ophthalmology, London, UK; 2 University of Utah, Salt Lake City, UT USA

**Keywords:** Predictive markers, Outcomes research

## Abstract

This article provides a comprehensive overview of clinical trial design and regulatory pathways essential for drug development, specifically in the context of retinal diseases. Key concepts include trial structure, efficacy and safety endpoints, and regulatory expectations from agencies like the FDA. It delves into recent regulatory advancements, such as the inclusion of low-luminance vision as a secondary endpoint and analyses case studies from age-related macular degeneration (AMD) trials. Approvals for key retinal drugs, such as ranibizumab and aflibercept, treatments for AMD and diabetic macular oedema, are discussed highlighting criteria like the 15-letter gain/loss in visual acuity as approvable/clinical meaningful efficacy endpoints. Insights into geographic atrophy (GA) and diabetic retinopathy trials showcase the evolving landscape, where anatomical endpoints and new drugs bring fresh challenges and opportunities. It also emphasizes the importance of academic-industry collaboration, citing instances of gene therapy development and innovative endpoint measures like the Multi-Luminance Mobility Test for retinal dystrophies. The overarching aim of this lecture was to demystify the process that spans the design of clinical trials to regulatory approval of drugs so that clinicians understand these complexities. In particular, it is important to understand the reasons behind selection of trial design, inclusion and exclusion criteria, primary and secondary efficacy endpoints and safety endpoints. Since this lecture, there have been important changes in this field including new guidance from the Food and Drug Administration (FDA) as well as lessons learnt from recent drug approvals that are included in this manuscript.

## Understanding the rules of engagement with the FDA and similar regulatory agencies

For a drug to be approved by the FDA or other regulatory agencies, the pivotal study must define a primary efficacy endpoint which shows a clinically meaningful benefit in a well-controlled study with adequate statistical power. In a common disease, two parallel studies are planned, which are usually similar in design. The primary endpoints and/ or the comparator used in the trials can be slightly different but to gain approval, the results from both studies need to be positive.

## Wet AMD trials

In retina, only a handful of endpoints have been used for drug approval [1]. The most commonly used is the change of best corrected visual acuity (BCVA). It can be the proportion of patients with a 15-letter gain or prevention of a 15-letter loss in the treated group vs the control group. For instance, ranibizumab was approved for neovascular age-related macular degeneration (nAMD) based on the proportion of eyes that lost less than 15 letters. In the ranibizumab group, 91–98%, met this endpoint compared with 60–66% of the control group over 12 months [2].

For diabetic macular oedema (DMO), the endpoint was the proportion of eyes that gained 15 letters. For the ranibizumab arms, 34–45% achieved this endpoint as compared to 12–18% in the control groups over 2 years [3]. The reason that 15 letters was chosen is that this change is considered clinically meaningful and also not likely to occur due to measuring errors. A study in Moorfields showed that a patient can have a six to seven letters difference in BCVA ETDRS score on the same day by different certified visual acuity assessors [4]. This should not be confused with the often-reported mean visual gains of about 10 letters improvement in patients treated with anti-VEGF in wet AMD and more often in DMO patients. These are easier to understand but were not used as approvable primary endpoints until recently. The aflibercept approval for DMO was based on non-inferiority of the proportion of eyes that did not lose more than 15 letters compared to ranibizumab, which was achieved by about 95% in both aflibercept and ranibizumab groups [5].

The concept of non-inferiority for comparing treatment with different anti-VEGF agents for wet AMD using mean BCVA changes from baseline was probably first introduced in the CATT study [6] and also adopted in the IVAN study [7]. The statistical concept of non-inferiority came from the generic and biosimilar world where equivalence has to be proven to the originator drug. It is beyond the scope of this paper to discuss the principle in detail, but non-inferiority using mean change from baseline in BCVA letter score has only been used in the most recent approvals, of brolucizumab, faricimab and aflibercept high dose. In a recent FDA draft guidance on neovascular AMD development, a 4.5 letter margin was set against on label ranibizumab or aflibercept [8].

It is important to understand that if superiority study design is to be used in the future, it might be insufficient to show statistical significance of superiority alone, there is also a need for the change to be clinical meaningful. For the FDA, that would be 15 letters of BCVA. In other words, if “mean change from baseline” is used, the study drug has to be 15 letters superior to the comparator. The alternative would be to have a statistically significant proportion of patients with 15 letters loss or gain. In wet AMD, if the new drug is to be compared with the current standard of care (aflibercept 2 mg being the most common anti-VEGF comparator), it would be challenging for many patients to have 15 letters loss on aflibercept 2 mg, so it would be likely that the sponsor (drug company) would go for an endpoint of 15 letter gain. However, to achieve 15 letters gain, further considerations need to incorporated to the eligibility criteria. For example, it would be challenging to achieve 15-letter gain if the baseline vision is good, so the inclusion criteria need to be restricted to only allowing patients with poorer baseline vision that have a better chance to obtain a 15-letter gain. Nonetheless, there are caveats in this assumption as patients with poorer baseline vision might also have irreversible structural changes such as fibrosis that would not permit a gain in BCVA.

However, the FDA has clarified that the new drugs do not need to be compared against the standard of care. In fact, a new drug could be compared to no treatment, sham or a much lower dose of the new drug (which is practically non-effective). In wet AMD, that would be difficult as vision loss is rapid and probably not reversible after a period of no treatment. Furthermore, the study period for wet AMD for approval is usually at least 9 months, so abstaining from treatment in this potentially blinding condition would raise ethical issues in conducting such a study. There are ways to get around this, by adding rescue therapy. Rescue therapy means if the patient has lost significant amounts of vision, the investigator can then treat the patient with standard of care (an approved anti-VEGF agent). However, this leads to variations, as the amount of vision loss that is acceptable to one investigator could be very different from another. In addition, if the primary endpoint is the proportion of patients with 15 letters loss, then the data could be messy when different investigators apply rescue treatment to patients at varying levels of vision loss before a 15-letter loss occurs.

It is also acceptable for BCVA to be measured in different light conditions. Recently, low luminance VA (LLVA) has been studied and considered at least as a secondary endpoint. LLVA is the BCVA after refraction with a 2+ neutral density filter placed in front of the patient’s eye. In general, LLVA is lower than BCVA, and the difference between BCVA and LLVA is called the low luminance deficit (LLD). The importance of LLD is not clear but there are some data to suggest that it has a potential in the prediction of treatment outcomes [9]. Therefore, if a drug developer believes that their drug can improve LLVA without improving BCVA, the FDA appears to be willing to accept that as the primary efficacy endpoint, providing the difference in outcome between the trial arms is 15 or more letters.

In addition to efficacy requirements, the FDA has also provided safety guidance for wet AMD trials. This being that at least 300 patients with safety data should have been treated to the primary endpoint (usually more than 9 months) at the intended marketed dose or higher. This means that the sample size needs to consider drop-outs to obtain a final sample of at least 300. This safety data does not include patients who were treated with a lower dose in the study. In addition, there should be “enough” patients treated for 2 years, the exact number is not given. This explains why there are parallel Phase 3 clinical trials on an intervention that report primary outcomes at 9–12 months. At least one of these two studies has to continue for 2 years and have a final sample size of 300 patients. This guidance is limited to small molecules and biologics. The rules for gene therapy are also different, and the duration of safety data collection is likely to be much longer.

## Geographic atrophy

In 2022, at the time of this lecture, two anti-complement trials were undergoing regulatory approval [10]. At that time, the FDA had agreed in principle to the use of differences in the slope of the mean rate of GA lesion growth as an approved endpoint. At the time of writing this manuscript in September 2024, anti-C3 had been approved by the FDA, but not by the European Medicines Agency (EMA). It is also interesting that both anti-C3 and anti-C5 were given a label of ‘geographic atrophy’ despite the anti-C3 trials including both foveal and non-foveal involving lesions whilst in the anti-C5 trials, only non-foveal involving lesions were included. This has significant implications for future GA study designs. Foveal involving lesions have less opportunity for visual change, so only including non-foveal lesions in a study means a better chance of showing functional / vision benefit (i.e. less visual loss over time). Based on the anti-C3 trials, the treatment benefit of anti-C3 appears to be higher in non-foveal involving lesions, partly explained by the slower growth rate of foveal involving lesions.

In drug development, being the first in class and particularly the first ever treatment for a condition, is very challenging. However, compounds that are considered to be followers have the advantage of learning from the lead molecule. It is not uncommon that the third molecule in the class ends up being the market leader, an example being aflibercept, the third anti-VEGF approved for retinal vascular diseases.

## Diabetic retinopathy

Diabetic retinopathy (DR) is another retinal condition with an FDA-approved anatomical endpoint. The diabetic retinopathy severity scale (DRSS) score [11] was used for approval of intravitreal (IVT) anti-VEGF injections for the treatment of DR by the FDA. The proportion of eyes with a 2 or more step improvement of DRSS score was used for the approval of ranibizumab [12] and aflibercept [13]. It was somewhat surprising that faricimab did not receive approval for DR based on their DMO study. One interpretation is that there is no agreed non-inferiority margin accepted by the FDA for DRSS, and faricimab did not show superiority to a control group but rather showed similarity to aflibercept in their pivotal DMO studies [14]. Similarity does not equate to non-inferiority. Recently, a company suggested that for an oral compound, a person level (combining the scoring of 2 eyes) 3 step DRSS score deterioration can also be accepted by FDA. This unsurprising as this was already used by an oral Protein Kinase C inhibitor (ruboxistaurin) some years ago [15]. Currently, anti-VEGF agents are not approved for DR in Europe other than ranibizumab for proliferative diabetic retinopathy (PDR). The exact reason for this decision is not clear. The PDR approval might have been based on data from DRCR.net Protocol S [16], which showed some improvement of visual function. For DR in general, regulatory authorities in Europe might be looking for different endpoints rather than a 2 steps DRSS improvement.

Returning to the discussion on study design, although ranibizumab and aflibercept are approved for diabetic retinopathy in the US, for a new drug, the sponsor can conduct a superiority study against no treatment or sham treatment or placebo. This is more acceptable to the community as despite ranibizumab and aflibercept being approved for this condition, in real-life, their use for this indication is low. Therefore, the standard of care for this condition is still observation despite the approval of these anti-VEGF agents. It would not be an issue in countries that have not approved their use for diabetic retinopathy.

It is also important to understand that the new drug does not need to be better than historic efficacy data of ranibizumab or aflibercept. It has to be statistically significantly and clinical meaningfully better than the comparator (sham treatment or placebo). Clinically meaningful has been established by ranibizumab and aflibercept but the definition of the minimum level of efficacy based on DRSS score that would be acceptable is unclear.

Other considerations such as the invasiveness of the treatment or the adverse event rate of the treatment may also need to be taken into account. For instance, gene therapy given through one single suprachoroidal injection might be different from another intravitreal drug given every 8 weeks. Similarly, oral therapy may have different endpoints. Although oral compounds might appear attractive to ophthalmologists, effective oral compounds often have significant risks of systemic adverse events. Finally, the convenience of oral treatment for patients also needs to be compared to repeated intravitreal injections, which although relatively safe, have a large treatment burden for both patients and the health care system. Topical therapy such as eyedrops could potentially provide a good solution and ophthalmologists look forward to such advancements in retinal diseases.

## Masking to eliminate bias

It is common practice in almost all clinical trials for oral or topical treatments to use a placebo. However, for intravitreal development, it is considered unethical to perform an intravitreal injection with a placebo or saline. Recently, the FDA stated [17] that an intravitreal sham injection is not generally considered good enough for masking as the agency believes patients can tell the difference between an intravitreal injection and a sham injection. The exact reason for this decision is unknown as intravitreal sham injections have been standard for most intravitreal retinal drug approval studies including the most recent approvals of faricimab and high-dose aflibercept 8 mg. It is probably true that patients might have some visual disturbance when the drug is injected into the eye.

In subretinal gene therapy, there is no masking as it is unethical to perform surgery without injecting the gene. Similarly, when injecting intravitreal small molecules within bioerodible or bioresorbable implants, the patients can potentially see the implant or experience floaters. It is challenging to understand the issue of masking in these cases. It will be interesting to see how regulatory agencies handle these approvals when the time comes.

## Uveitis

The approval of intravitreal dexamethasone implants for uveitis was based on the percent of patients reaching a vitreous haze score of 0 (i.e. no inflammation) [18]. At week 8 this was 47% vs 12% in the control group. In addition, the percentage of patients achieving a BCVA improvement of 15 letters from baseline was 43% vs 7% in the control group. The FDA, in general, accepts 2 steps reduction of vitreous haze score, so theoretically from score 3 to score 1. However, it might be difficult to recruit a lot of patients with vitreous haze score more than grade 2. In recent communication, the FDA was willing to accept patients achieving a reduction of vitreous haze score to 0, if all included patients have a vitreous haze score of at least 1.5. The study endpoint is week 8 but addition safety data is likely to be needed.

## MacTel and EZ loss

Using anatomical endpoints for drug approval is gaining more traction. Encapsulated cell therapy producing CNTF implant (NT-501), in two Phase 3 studies, has demonstrated the rate of change of ellipsoid zone (EZ) loss as the primary endpoint in macular telangiectasia (MacTel) Type 2 [19]. At the time of writing, it is not yet approved by the FDA. Nonetheless, if it were to be approved, this would mean another anatomical endpoint could potentially be used for other retinal diseases when the central vision or BCVA might not be affected until late in the disease process. There is a suggestion that EZ loss might be acceptable for intermediate AMD.

In addition, the FDA had suggested that the onset of nAMD or GA can be potentially used as an approvable endpoint, but so far this approach has yet to be tried. The main reason is probably the relatively low event rate. On average, the conversion to nAMD of the fellow eye of patients with unilateral nAMD is about 10% per year [20]. The rate of conversion to GA is even lower. Nonetheless, using OCT and AI, we might be able to improve the prediction. However, if the accuracy of prediction is improved, it remains to be seen whether eyes with high conversion rates are eyes that might be too advanced for treatment or too rare to find. For instance, Lad and colleagues [21], identified one subgroup of GA with a 75% risk of converting to GA in 1 year but this group comprises only 1% of the study population. When we consider that this study used images from participants in the AREDS2 study [22], which only included patients with large drusen and/or pigmentary changes, the proportion of these patients in the whole population with intermediate AMD population is likely to be even lower.

## Collaboration between academia and industry is critical

Lastly, collaboration between academia and industry is important to take novel interventions through the approval process to translation to clinical practice. As an example, Nightstar was born out of academics and was bought by Biogen for 810 million US dollars. It was a time of triumph, however, both programs subsequently failed the pivotal study despite showing positive trends on secondary endpoints. This resulted in Biogen deprioritising Ophthalmology and the development of those assets were terminated [23]. So why would a promising research project such as this fail to get to patients?

The first gene therapy in the history of the FDA was for a retinal inherited disease (RPE65 mutation). The approval of this gene therapy for RPE65 mutation-associated retinal dystrophy was based on the multi-luminance mobility testing (MLMT) score change from baseline to year 1 as an endpoint [24]. The MLMT [25] was designed to measure changes in functional vision, as assessed by the ability of a subject to navigate a course accurately and at a reasonable pace at different levels of environmental illumination. The MLMT was assessed using both eyes and each eye separately at one or more of seven levels of illumination, ranging from 400 lux (corresponding to a brightly lit office) to 1 lux (corresponding to a moonless summer night). Each light level was assigned a score code ranging from 0 to 6. A higher score indicated that a subject was able to pass the MLMT at a lower light level. A score of −1 was assigned to subjects who could not pass MLMT at a light level of 400 lux. The MLMT of each subject was videotaped and assessed by independent graders. The MLMT score was determined by the lowest light level at which the subject was able to pass the MLMT. The MLMT score change was defined as the difference between the score at baseline and the score at Year 1. A positive MLMT score change from baseline to Year 1 visit indicated that the subject was able to complete the MLMT at a lower light level [26]. This is a combination of assessing improvements in visual acuity and visual field in different light intensity allowing a clinically meaningful benefit to be demonstrated in a controlled but pseudo-real-life activity.

However, in the Biogen/Nightstar study, the retinal sensitivity improvement using microperimetry was chosen as the primary efficacy endpoint, which is much more difficult to demonstrate, in particular, the level of gain needed by the agency (7 dB on average and in at least pre-defined 5 points). It is therefore important to understand both the regulatory requirements as well as the ability to achieve a primary endpoint within the time frame of the study period (which is usually one or 2 years).

Functional endpoints are often difficult to demonstrate, particularly in patients with highly variable vision and/or using a test such as microperimetry, which are challenging to perform consistently.
